# Elucidating the Histone Deacetylase Gene Expression Signatures in Peripheral Blood Mononuclear Cells That Correlate Essential Cardiac Function and Aid in Classifying Coronary Artery Disease through a Logistic Regression Model

**DOI:** 10.3390/biomedicines11112952

**Published:** 2023-11-01

**Authors:** K. Monisha, S. Mahema, M. Chokkalingam, Sheikh F. Ahmad, Talha Bin Emran, Paramasivam Prabu, Shiek S. S. J. Ahmed

**Affiliations:** 1 Drug Discovery and Multi-omics Laboratory, Faculty of Allied Health Sciences, Chettinad Academy of Research and Education, Chettinad Hospital and Research Institute, Kelambakkam 603103, India; 2 Department of Cardiology, Chettinad Hospital and Research Institute, Chettinad Health City, Kelambakkam 603103, India; 3 Department of Pharmacology and Toxicology, College of Pharmacy, King Saud University, Riyadh 11451, Saudi Arabia; 4 Department of Pathology and Laboratory Medicine, Warren Alpert Medical School, Brown University, Providence, RI 02912, USA; 5 Legorreta Cancer Center, Brown University, Providence, RI 02912, USA; 6 Department of Pharmacy, Faculty of Allied Health Sciences, Daffodil International University, Dhaka 1207, Bangladesh; 7 Madras Diabetes Research Foundation, Chennai 600086, India; 8 Department of Neurology, University of New Mexico Albuquerque, Albuquerque, NM 87131, USA

**Keywords:** *HDAC* profiling, atherosclerosis, epigenetic biomarkers, coronary artery disease, left ventricular structural indices

## Abstract

A proinflammatory role of *HDACs* has been implicated in the pathogenesis of atherosclerosis as an emerging novel epigenetic diagnostic biomarker. However, its association with the clinical and cardiovascular function in coronary artery disease is largely unknown. The study aimed to profile the gene expression of *HDAC1*–*11* in human peripheral blood mononuclear cells and to evaluate their influence on hematological, biochemical, and two-dimensional echocardiographic indices in CAD. The *HDAC* gene expression profiles were assessed in 62 angioproven CAD patients and compared with 62 healthy controls. Among the *HDACs*, upregulated *HDACs 1,2*, *4*, *6*, *8*, *9*, and *11* were upregulated, and *HDAC3* was downregulated, which was significantly (*p* ≤ 0.05) linked with the hematological (basophils, lymphocytes, monocytes, and neutrophils), biochemical (LDL, HDL, and TGL), and echocardiographic parameters (cardiac function: biplane LVEF, GLS, MV E/A, IVRT, and PV S/D) in CAD. Furthermore, our constructed diagnostic model with the crucial *HDACs* establishes the most crucial *HDACs* in the classification of CAD from control with an excellent accuracy of 88.6%. Conclusively, our study has provided a novel perspective on the *HDAC* gene expression underlying cardiac function that is useful in developing molecular methods for CAD diagnosis.

## 1. Introduction

Coronary artery disease (CAD) continues to be a leading cause of mortality globally [1]. CAD is caused by atherosclerosis. Plaque formation and progression result from the intricate interaction of cellular molecules, exacerbated by traditional risk factors such as diabetes, smoking, hypertension, and obesity. These traditional risk factors significantly impact the endothelium and increase the disease burden, resulting in significant complications [2]. Clinical symptoms, an electrocardiogram (ECG) [3,4], stress testing, cardiac computed tomography (CT), and molecular markers [5] are used to diagnose coronary artery disease. Despite these diagnostic methods, there is no effective early detection procedure for CAD. Consequently, a diagnostic method that can screen for and predict CAD with high sensitivity and specificity is still desired.

Recent discoveries on epigenetic regulation implicate them as predictive markers in clinical contexts for cancer, neurodegenerative disorders, cardiovascular diseases, metabolic syndrome, inflammation, and immune disorders [6]. Several epigenetic modifications, such as histone protein modification, DNA methylation, microRNA expression, and long noncoding RNAs, influence transcription machinery, resulting in cellular and molecular changes [7]. Histone deacetylase (*HDAC*) is essential in modulating cellular gene expression and contributing to physiological changes in the cardiovascular system [8]. Several animal and cell model experiments implicate *HDACs* in endothelial and inflammatory processes associated with the pathogenesis of CAD [9]. However, the effect of *HDACs* on the structure and function of the myocardium is largely unknown. Establishing the *HDAC* mechanisms associated with cardiac physiology may aid in developing a biomarker for diagnosing CAD.

This study aimed to profile the *HDAC* gene expression in human peripheral blood mononuclear cells (PBMCs) to correlate with the hemodynamic status of the heart based on the hematological, biochemical, and two-dimensional echocardiographic indices in participants with CAD. The assessment of hemodynamic status reveals a substantial epigenetic connection between *HDACs* and cardiovascular function. Further, the developed diagnostic model with logistic regression provides evidence to rationalize specific HDACs as a possible diagnostic marker for CAD.

## 2. Materials and Methods

### 2.1. Clinical Specimens

The institutional human ethics committee of the Chettinad Academy of Research and Education (IHEC/10-17/Proposal No. 372) was obtained for this study. All participantssigned informed consent before the initiation of the investigation process. Study participants were recruited at Chettinad Super Specialty Hospital between August 2019 and December 2019 and underwent a coronary angiogram with angina symptoms. Each study participant’s demographic and clinical histories were recorded based on a questionnaire approved by the institution’s institutional review board. By the inclusion and exclusion criteria, 124 participants were recruited for the study. The inclusion criteria for the study participants include the following:(1) Participants belonging to South Indian ancestry of both genders. (2) Physiological status was diagnosed or classified by a qualified senior consultant cardiologist based on the coronary angiogram (for CAD, a lesion greater than 30% in the primary coronary artery or main branches, and participants without CAD, lesions below 30%). The exclusion criteria include the following: 1. previous history of myocardial infarction (MI); 2. heart failure; 3. arterial revascularization; 4. rheumatic disease; 5. cardiomyopathies; 6. pericardial diseases; 7. a severe systemic inflammatory disorder; and 8. acute or chronic respiratory diseases.

### 2.2. Human Anthropometric and Biochemical Evaluation

Standardized methodologies were used to measure anthropometric measurements such as height (m2) and weight (kg) to yield the body mass index (BMI). A mercury sphygmomanometer was used to measure systolic blood pressure (BP) and diastolic BP for the participants. The biochemical indices such as glycated hemoglobin (HbA1c) (urease method), fasting (FBS) and postprandial blood glucose (PPBS) (hexokinase method), total cholesterol (T. cholesterol) (CHOD/POD end method), serum triglycerides (TGL) (GPO POD method), high-density lipoprotein (HDL) (direct method), low-density lipoprotein (LDL) (Friedewald formula), and complete blood count including, total leukocyte count (TLC), red blood cell count (RBC), hemoglobin (HB), packed cell volume (PCV), mean corpuscular volume (MCV), mean corpuscular hemoglobin (MCH), mean corpuscular hemoglobin concentration (MCHC), red cell distribution width (RDW), platelets, basophils, eosinophils, lymphocytes, monocytes, and neutrophils were assessed using Coulter LH 780 Hematology Analyzer (Beckman Coulter, Brea, CA, USA).

### 2.3. Gene Expression Profiling of HDAC

Peripheral blood (5 mL) was collected from the study participants (62 CAD and 62 control) in the K2EDTA BD Vacutainer (BD Vacutainer^®^) at Cath ICU, Department of Cardiology, Chettinad Super Specialty Hospital. Following the manufacturer’s protocol, histopaque (Cat#1077, Sigma-Aldrich, St. Louis, MO, USA) was used to isolate the PBMCs from the collected whole blood. Total RNA was extracted from PBMCs (1.30 × 10^6^ cells/mL) using Trizol reagent (cat#15596026, Invitrogen, Waltham, MA, USA). The Nanodrop 2000 Spectrophotometer (Thermo Scientific, Waltham, MA, USA) was used to quantify the collected RNA. The reverse transcription was carried out for cDNA synthesis using a SuperScript III First-Strand (Life Technologies, Carlsbad, CA, USA). The gene expression was performed using ABI-7000 (Applied Biosystems, Waltham, MA, USA) with SYBR green master mix (Takara, Kusatsu, Japan) and gene-specific primers for *HDACs* (Table 1). The *HDAC* gene expressions were normalized with the housekeeping gene (*GAPDH*), and the expression was calculated by the ΔΔCt method.

### 2.4. Echocardiography Imaging

Transthoracic 2D echocardiography was performed using Esaote (MyLabTM25Gold) according to the guidelines of the American Society of Echocardiography [10,11]. Left ventricular structural indices such as left ventricular interventricular septal, posterior wall thickness, left ventricular end-systolic dimension, and left ventricular end-diastolic dimension were measured using M-mode echocardiography at the chordae tendineae level to determine left ventricular ejection fraction (biplane LVEF). Further, the mass index of the left ventricle (LVMI) was calculated using the Devereux 1987 method. The biplane Simpson’s technique and global longitudinal strain (GLS) were utilized to determine the left ventricle’s systolic functions. Furthermore, pulsed wave transmitral flow and tissue Doppler velocity were implemented to determine the left ventricular diastolic functions based on early and late transmitral flow velocity, mitral inflow E/A ratio (MV E/A), mitral peak lateral (L E/e′) and septal myocardial early diastolic velocity (S E/e′), isovolumetric relaxation time (IVRT), pulmonary vein AR duration (PV AR), and pulmonary venous systolic velocity/diastolic velocity ratio (PV S/D).

### 2.5. Diagnostic Model Using Binary Logistic Regression

To assess the diagnostic value of the *HDACs* related to anthropometric, biochemical, and imaging parameters, the diagnostic model was constructed by fitting the expression value of the significant *HDACs* into a binary logistic regression model (glm2 package version 1.2.1, R package). Youden’s J index was used to find the optimal threshold. All 124 participants were classified into training (70%) and test set (30%). The training cohort was utilized for constructing the model, while the testing cohort was employed to assess the model’s performance. Then, the receiver operating characteristic (ROC) curves were utilized to evaluate the model’s efficacy.

### 2.6. Statistical Examination

The characteristics of participants, such as anthropometric, biochemical, molecular, and imaging parameters, were represented as the mean ± standard deviation. The difference between the CAD and control was determined using a student *t*-test for each characteristic except gender data. The categorical data (gender) were assessed throughthe Chi-square test. The Pearson correlation was implemented to assess the association between the characteristics. All statistical analyses were performed using SPSS (version 21), and significance was determined if the *p*-value was less than 0.05.

## 3. Results

### 3.1. Evaluation of Anthropometric, Hematological, and Biochemical Features

In anthropometric measurements, systolic BP and diastolic BP were significantly elevated in CAD. Similarly, lipid parameters such as T. cholesterol (*p* < 0.05), TGL (*p* < 0.001), and LDL (*p* < 0.001) were significantly elevated in CAD compared to the control group. In contrast, HDL (*p* < 0.001) decreased in CAD compared to the controls. Similarly, the complete blood count parameters, including TLC (*p* < 0.001), HB (*p* < 0.001), RBC (*p* < 0.001), PCV (*p* < 0.001), basophils (*p* < 0.05), lymphocytes (*p* < 0.05), monocytes (*p* < 0.001), and neutrophils (*p* < 0.001) were significantly altered. In contrast, there were no statistically significant differences between CAD and the control for age, BMI, FBS, PPBS, MCV, MCH, MCHC, RDW, platelets, eosinophils, and HbA1c levels (Table 2).

### 3.2. Gene Expression Profiling of HDACs in PBMCs

The gene expression of *HDACs* was evaluated in PBMCs and compared between CAD and control groups. As shown in Table 3, CAD exhibited a significant increase in *HDAC1*, *HDAC2*, *HDAC4*, *HDAC6*, *HDAC8*, *HDAC9*, and *HDAC11* expression, while *HDAC3* expression decreased compared to the control. In contrast, *HDAC5*, *HDAC7*, and *HDAC10* failed to reach the minimal significance level between groups.

### 3.3. Assessment of 2D Echocardiographic Imaging

Using echocardiography, the structure and functional behavior of the heart were evaluated in 124 participants. Table 4 displays the outcome of echocardiographic indices. In CAD, the echocardiographic indices of LVMI, PV S/D, and IVRT were significantly increased. In contrast, the ratios of MV E/A, S E/e′, L E/e, PV AR, biplane LVEF, and GLS weredecreased compared to the control. Consequently, the statistical analysis of echocardiographic indices confirms the left ventricular dysfunction in CAD relative to healthy individuals.

### 3.4. Correlation of Significantly Altered Hematological and Biochemical Features Characteristics with HDACs

**Figure 1:**
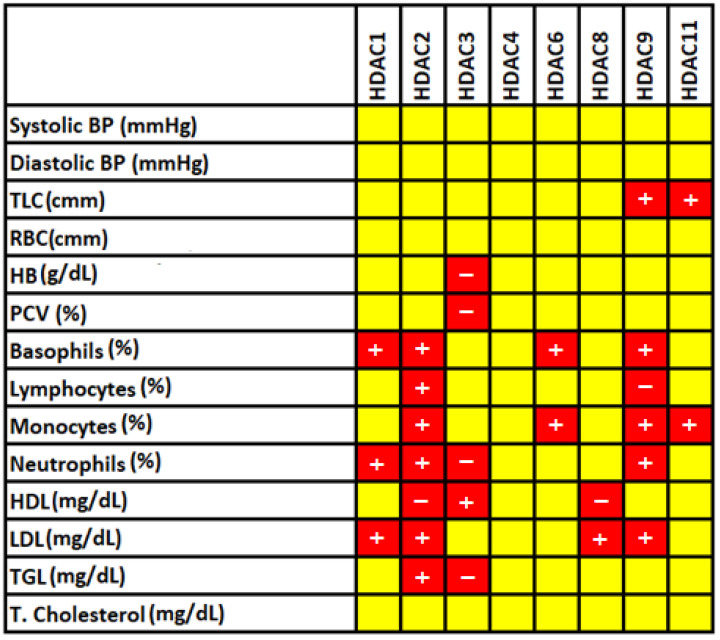
Correlation analysis of significantly altered hematological and biochemical features with *HDACs* (*1–4*, *6*, *8*, *9*, and *11*) in CAD participants. The yellow color represents an insignificant association between the analyzed features, and the red color indicates a significant association. Of those, the mathematical sign + and − represent positive and negative correlation, respectively.

The association between *HDACs* and anthropometric and biochemical characteristics was evaluated using Pearson correlation analyses. *HDAC2* shows a positive correlation with basophils (r = 0.59, *p* = 0.026), lymphocytes (r = 0.782, *p* < 0.001), monocytes (r = 0.677, *p* = 0.008), neutrophils (r = 0.703, *p* = 0.005), LDL (r = 0.883, *p* = <0.001), TGL (r = 0.643, *p* = 0.003), and negatively correlated with HDL (r = −0.773, *p* = 0.001). Further, *HDAC9* showed a positive correlation with TLC (r = 0.925, *p* < 0.001), basophils (r = 0.728, *p* = 0.017), monocytes (r = 0.778, *p* = 0.008), neutrophils (r = 0.753, *p* = 0.012), and LDL (r = 0.752, *p* = 0.012), and a negative association with lymphocyte (r = −0.685, *p* = 0.029). Likewise, Pearson correlation analysis revealed a significant relationship between *HDAC1*, *HDAC3*, *HDAC6*, *HDAC8*, and *HDAC11*, along with anthropometric and biochemical characteristics (Figure 1). In contrast, *HDAC4* has no significant relationship with anthropometric or biochemical parameters.

### 3.5. Correlation between HDAC Levels and 2D Echocardiographic Parameters

**Figure 2:**
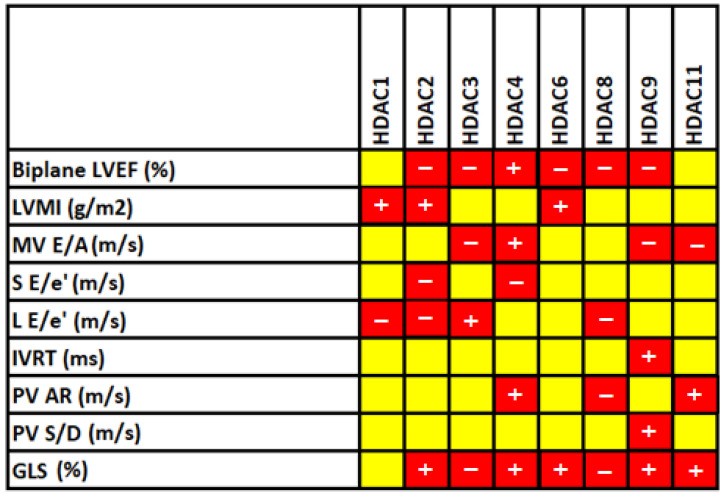
Correlation analysis of significantly altered echocardiographic indices with *HDACs* (*1–4*, *6*, *8*, *9*, and *11*) in CAD participants. The yellow color represents an insignificant association between the analyzed features, and the red color indicates a significant association. Of those, the mathematical sign + and − represent positive and negative correlation, respectively.

In correlation analysis of *HDACs* with echocardiographic indices, *HDAC2* showed a positive correlation with LVMI (r = 0.634, *p* = 0.015) and GLS (r = 0.709, *p* = 0.004) and a negative association with biplane LVEF (r = −0.701, *p* = 0.005), S E/e’ (r = −0.811, *p* < 0.001), and L E/e’ (r = −0.773, *p* < 0.001). Similarly, *HDAC9* expression confirmed the negative correlation with biplane LVEF (r = −0.8436, *p* = 0.003) and MV E/A (r = −0.837, *p* = 0.003) and the positive association of GLS (r = 0.793, *p* = 0.006), IVRT (r = 0.748, *p* = 0.013), and PV S/D (r = 0.697, *p* = 0.025). Likewise, a significant association was noticed for other *HDACs* with echocardiographic indices (Figure 2), presenting the involvement of *HDACs* with heart structure and functional indices.

### 3.6. Diagnostic Model with Crucial HADCs for CAD Classification

**Figure 3:**
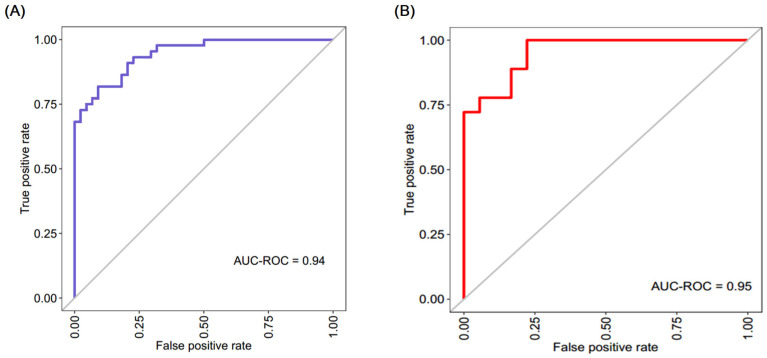
Area under the ROC curve graphs for the CAD diagnostic model’s training (**A**) and test (**B**) sets based on *HDAC* gene expression levels. The color code (blue and red) represent the area under the curve (AUC).

A logistic regression analysis was performed after the screening of *HDACs* based on the correlation significance with hematological, biochemical, and echocardiographic indices. The diagnostic model was created using the R-package version 3.2.0, incorporating the variables *HDAC1*, *HDAC2*, *HDAC3*, *HDAC4*, *HDAC6*, *HDAC8*, *HDAC9*, and *HDAC11*. The model utilizes a training set including 70% of the randomly selected 124 people and a test set consisting of the remaining 30%. The diagnostic regression equation was constructed as logit (P) = −16.937 + 0.763 × *HDAC11* + 1.595 × *HDAC9* + 0.298 × *HDAC8* + 1.652 × *HDAC4* − 0.676 × *HDAC3*. The diagnostic capabilities of this equation were evaluated using ROC analysis, as shown in the figures. The training set yielded an accuracy of 88.6% with a 0.94 area under the curve (AUC), a specificity of 86.4%, and a sensitivity of 90.9% (Figure 3A). Similarly, the test set demonstrated an accuracy of 83.3% with a 0.95 AUC, a specificity of 72.2%, and a sensitivity of 94.4% (Figure 3B). These results provide evidence that these *HDACs* possess strong predictive capabilities of CAD.

## 4. Discussion

The emphasis of our research has been to observe the types of epigenetic alterations in patients presenting with angina pectoris that are further grouped as angioproven CAD and healthy controls. The scientific application of this perception can immensely support the development of effective diagnostic biomarkers for CAD. Earlier clinical studies on animals and human atherosclerotic tissues have demonstrated the epigenetic modification that plays a predominant role in the regulatory network of inflammation, oxidative stress, and vascular smooth muscle cell proliferation, contributing to vascular diseases such as atherosclerosis and restenosis [12]. Evidence of *HDAC* modification in atherosclerosis has been well-established as a valid target for novel therapeutic approaches [13]. Yet, the increased understanding of *HDAC* regulatory activity in atherosclerosis as a diagnostic biomarker associated with echo imaging, biochemical, and hematological analysis is unseen. Hence, to our knowledge, the present study is the first report demonstrating *HDAC* profiling and altered gene expression in the PBMCs of patients with CAD and healthy controls. Our study demonstrated the significant variation in *HDACs 1*–*11* activity between the angioproven CAD and control individuals. Regarding HDAC profiling, our *t*-test analysis identified certain *HDACs* as the exclusive significant factor. Further, using the Pearson correlation, the significant *HDACs* and biochemical, hematological, and echocardiographic parameters were analyzed and proven to be possible diagnostic markers for CAD.

Histone modification profiling in human PBMCs was performed between angioproven CAD and healthy participants to discover a novel selective diagnostic marker for CAD. Interestingly, by profiling *HDACs 1*–*11*, we observed changes in the *HDAC* gene expression and their statistical significance in the PBMCs between CAD and healthy participants. Several animal, cell, and human model studies have highlighted that class I *HDAC* is predominant in mediating proinflammatory molecules, inflammation, and endothelial dysfunction and regulating VSMC proliferation, thrombus formation, and atherosclerosis [14]. In our study, Class I *HDACs* (*1-3*, and *8*) were solely significant and dysregulated in the study group. Studies by Manea et al. revealed that *HDAC1* and *2* were upregulated in human atherosclerotic aorta/carotid arteries, and ApoE¯/¯ mice thereby witnessed the dominant role of *HDAC1* and *2* in the development of atherosclerotic plaque [15]. Yao et al. have reported that the levels of class I *HDAC3* were downregulated, which legalizes the cyclic strain and promotes the migration and proliferation of VSMCs in the development of atherosclerosis [16]. Another study by Kee et al. demonstrated that the inhibition of *HDAC8* in a mouse model lowers blood pressure, reduces the aortic wall thickness, and increases vascular relaxation, resulting in the inhibition of inflammation that leads to atherosclerosis [17]. In our present study, we observed statistically significant upregulation of *HDAC1, 2,* and *8* and downregulation of *HDAC3* in CAD participants.

Furthermore, Class II *HDACs* (*4*, *5*, *6*, *7*, *9*, and *10*) were analyzed, resulting in upregulated expressions of histone acetylation, of which *HDACs 4*, *6*, and *9* were statistically significant among the study participants. Numerous studies [18,19,20,21] have proved that *HDAC4* promotes VSMC proliferation, migration, and atherosclerotic plaque formation. In addition, *HDAC4* was involved in VSMC proliferation, which plays a crucial role in vascular calcification [22] and the inflammatory response [23]. Likewise, increasing evidence has supported our study results that *HDAC6* plays a decisive role in endothelial dysfunction [24], oxidative stress [25], and inflammation [26] and has a protective role in promoting vascular homeostasis [27]. Among all the classical *HDACs*, *HDAC9* is the most well-studied individual subtype for its association with atherosclerosis in animal and human models. It has been reported that an increase in *HDAC9* gene expression in human internal carotid [28], plasma/coronary artery disease [29,30], the blood of patients with large atherosclerotic lesions [31], and plaque vulnerability [32] results in plaque formation and the development of atherosclerosis that further causes severe cardiac events. However, our study was in line with other studies in which the levels of *HDAC* were upregulated and highly significant among our CAD participants. Although the *HDACs* (*5*, *7*, and *10*) showed dysregulation in CAD, they were not statistically significant. Of note, *HDAC11* is the only member of Class IV that has been least explored and analyzed in the development of atherosclerosis in humans and animals. Our study showed that Class IV (*HDAC11*) levels were significantly upregulated among CAD participants. In contrast to our investigation, Zhang and Ge et al. and Yanginler and Logie et al. [33,34] demonstrated that *HDAC11* potentially treats atherosclerosis. Hence, these findings determine the dysfunction of *HDAC11* in the pathogenesis of atherosclerosis and CAD, which must be further studied in detail.

In addition to *HDAC* gene expression profiling, we have also analyzed the association of significantly altered *HDACs* (*1–4*, *6*, *8*, *9*, and *11*) with the hematological and biochemical parameters that play a vital role in the cascade of events that leads to atherosclerosis. Despite all these studies, the association between *HDAC* levels and hematological/biochemical parameters related to CAD has not been explored. In our present investigation, our data showed that dysregulated levels of *HDACs* such as *HDAC2* (basophils, lymphocytes, monocytes, neutrophils, HDL, and LDL), *HDAC3* (HB, PCV, neutrophils, HDL, and LDL), *HDAC9* (TLC, basophils, lymphocytes, monocytes, neutrophils, HDL, and LDL), *HDAC1* (basophils and neutrophils), *HDAC6* (SBP, DBP, basophils, monocytes, and T. cholesterol), *HDAC8* (HDL and LDL), and less known *HDAC11* was correlated with the hematological/biochemical parameters (TLC and monocytes) of CAD patients. Studies such as Dorneles et al. and Chi et al. provided good evidence for our data by demonstrating an imbalance of *HDAC2* expression levels in obese patients [35] and the role of *HDAC6* in cardiac dysfunction regulated by angiotensin II [36]. Hence, the present experimental association of dysregulated *HDACs* with the hematological and lipid profiles of CAD indicates the crucial pathological role of *HDACs* in atherosclerosis [37], which can potentially serve as a diagnostic biomarker for CAD with clinical benefits.

To address whether dysregulated *HDAC* gene expression distresses the left ventricular mass diastolic and systolic function, we validated the statistically significant profiled *HDACs* with 2D transthoracic echocardiographic imaging. Left ventricular mass index is recognized as one marker of cardiovascular risk in patients without CAD. Abdi-Ali et al. [38] and Kee et al. [39] ruled out the significant association of LVMI with cardiac hypertrophy and future cascades of cardiac events. Our data found a similar association between dysregulated *HDACs 1*, *2*, and *6* and LVMI in the CAD group, demonstrating the regulatory mechanism of *HDAC* activity in LV mass hypertrophy. Further, the assessment of LV function showed a significant association of dysregulated *HDACs 1–4*, *6*, *8*, *9*, and *11* with systolic and diastolic function. Several studies in CAD patients have elucidated that subclinical LV systolic and LV diastolic impairments are independent markers for predicting CAD. Our data results were in line with a few studies performed on both animal and human models by Kimbrough et al., Chen et al., and Jeong et al. [40,41,42] that illustrate that dysregulated *HDACs* promote endothelial dysfunction and contribute to the pathological process that leads to CAD and mediated cardiac events. Altogether with prior research, alterations in *HDAC* mRNA expression are detected in numerous CAD-associated cells, including endothelial cells, smooth muscle cells, and cardiomyocytes. Comparable patterns of gene expression were identified in peripheral blood mononuclear cells (PBMCs) that correlate with cardiac function in CAD. Considering the CAD association of these *HDACs*, we constructed a diagnostic model with significant *HDACs* that showed accuracy greater than 83% in both training and testing sets, which showed that our model had a strong clinical application value. To our knowledge, no existing study has developed a diagnostic model utilizing *HDAC* gene expression. Therefore, this diagnostic model holds significant novelty and potential for application in laboratory settings. Overall, this study has demonstrated that the process of histone deacetylation has the potential to function as a diagnostic biomarker for coronary artery disease.

## 5. Conclusions

In conclusion, our study evaluated the gene expression levels of various classes of *HDACs* in the human peripheral mononuclear cells in patients with CAD and compared them with the healthy control group. It was observed that significant alterations in *HDACs 1*-*4*, *6*, *8*, *9*, and *11* were associated with the critical hematological, biochemical, and cardiac indicators of CAD. Further utilizing the *HDACs*, the diagnostic model was generated, which showed significant accuracy in classifying CAD from healthy normal.

## Figures and Tables

**Table 1:** List of primers used in our study.

S.No	Gene	FORWARD 5′<-----Sequence----->3′	REVERSE 5′<-----Sequence----->3′
1	*HDAC1*	GAGATGACCAAGTACCACAGC	TGACAGAACTCAAACAGGCC
2	*HDAC2*	TGACAAACCAGAACACTCCAG	TGACAAACCAGAACACTCCAG
3	*HDAC3*	GGACTTCTACCAACCCACG	CAGCACGAGTAGAGGGATATTG
4	*HDAC4*	ACAAGGAGAAGGGCAAAGAG	GCGTTTTCCCGTACCAGTAG
5	*HDAC5*	TCACCGCAAAACTCCTACAG	AGTTCCCGTTGTCATAGCG
6	*HDAC6*	TTCAACTCTGTGGCTGTGG	GGGACACATATAGCACACTGG
7	*HDAC7*	GCAGATCATTCAACAGCCATG	TTGGTAGAAGGTTTGCTGGG
8	*HDAC8*	AATTAACTGGTCTGGAGGGTG	TGCAGATCCAAATCCACGTAG
9	*HDAC9*	ACACATTACCAGGAGCACAAG	CAACATTTCCATCCTTCCGC
10	*HDAC10*	GTCCTTTACTTCTCCTGGCAC	GTAGTCAGCGTTTCCCATCC
11	*HDAC11*	GTTTCTGTTTGAGCGTGTGG	GGTAGATGTGGCGGTTGTAG
12	*GAPDH*	TGTCATCAACGGAAAGGC	GCATCAGCAGAAGGAGCA

**Table 2:** Characteristics of the study population in anthropometric, hematological, and biochemical features.

Parameters	Control (Mean ± Standard Deviation)	CAD (Mean ± Standard Deviation)	*p*-Value
Age (years)	44.90 ± 8.96	48.23 ± 9.34	0.096
Gender (male/female)	38/24	33/29	0.360
BMI (kg/m^2^)	23.76 ± 2.98	25.02 ± 1.19	0.081
Systolic BP (mmHg)	103.44 ± 18.43	118.65 ± 18.64	<0.001
Diastolic BP (mmHg)	69.62 ± 8.87	78.60 ± 14.02	<0.001
FBS (mg/dL)	99 ± 16.59	107.55 ± 12.18	<0.071
PPBS (mg/dL)	109.62 ± 38.86	105.34 ± 26.84	<0.065
TLC (cmm)	7034.88 ± 1700.96	9783.72 ± 4165.04	<0.001
RBC (cmm)	5.14 ± 0.65	4.62 ± 0.58	<0.001
HB (g/dL)	14.57 ± 1.62	13.18 ± 1.60	<0.001
PCV (%)	44.48 ± 4.96	40.05 ± 4.87	<0.001
MCV (fl)	87.23 ± 7.86	86.57 ± 7.11	0.686
MCH (pg)	28.66 ± 2.86	28.69 ± 2.84	0.955
MCHC (g/dL)	32.82 ± 0.68	33.12 ± 0.95	0.096
RDW (%)	14.25 ± 1.50	14.41 ± 1.54	0.631
Platelet (cmm)	2.83 ± 0.68	2.81 ± 0.73	0.926
Basophil (%)	0.57 ± 0.44	0.83 ± 0.49	0.015
Eosinophil (%)	3.66 ± 3.24	3.9 ± 3.62	0.750
Lymphocyte (%)	29.69 ± 8.79	36.03 ± 12.23	0.007
Monocyte (%)	4.18 ± 1.80	7.12 ± 2.86	<0.001
Neutrophil (%)	54.83 ± 8.77	66.46 ± 12.76	<0.001
HDL (mg/dL)	38.11 ± 7.00	28.81 ± 7.50	<0.001
LDL (mg/dL)	115.86 ± 27.92	165.83 ± 46.59	<0.001
TGL (mg/dL)	118.90 ± 65.48	189.90 ± 43.70	<0.001
Total Cholesterol (mg/dL)	182.302 ± 33.94	211.93 ± 52.79	0.003
HbA1c (%)	5.05 ± 0.43	5.35 ± 0.16	0.831

*p* < 0.05, statistical *p*-value < 0.05 considered as significant.

**Table 3:** *HDAC* gene expression of study participants.

*HDACs*	Control (Mean ± Standard Deviation)	CAD (Mean ± Standard Deviation)	*p*-Value
*HDAC1*	2.33 ± 1.21	2.91 ± 1.48	0.030
*HDAC2*	4.35 ± 1.69	5.51 ± 1.52	0.014
*HDAC3*	3.82 ± 0.73	2.28 ± 0.59	0.044
*HDAC4*	3.43 ± 0.98	4.52 ± 1.01	0.032
*HDAC5*	1.91 ± 0.37	3.48 ± 1.75	0.202
*HDAC6*	2.26 ± 0.70	2.52 ± 0.71	0.042
*HDAC7*	0.95 ± 0.17	1.58 ± 0.92	0.130
*HDAC8*	3.80 ± 2.36	5.21 ± 3.02	0.004
*HDAC9*	3.54 ± 1.99	6.25 ± 1.16	<0.001
*HDAC10*	0.84 ± 0.52	1.34 ± 0.55	0.184
*HDAC11*	3.36 ± 0.21	4.93 ± 2.19	<0.001

*p*-value < 0.05 considered as significant.

**Table 4:** Characteristics of the study population in echocardiographic parameters.

Echo Parameters	Control (Mean ± Standard Deviation)	CAD (Mean ± Standard Deviation)	*p*-Value
Biplane LVEF (%)	58.66 ± 1.77	48.51 ± 5.62	<0.001
LVMI (g/m^2^)	69.95 ± 25.91	83.97 ± 18.74	0.004
MV E/A (m/s)	1.13 ± 0.30	0.59 ± 0.56	<0.001
S E/e′ (m/s)	8.69 ± 0.97	7.08 ± 1.48	<0.001
L E/e′ (m/s)	10.20 ± 0.98	7.99 ± 1.05	<0.001
IVRT (ms)	69.54 ± 11.62	99.46 ± 16.33	<0.001
PV AR (m/s)	33.37 ± 7.88	28.14 ± 5.79	<0.001
PV S/D (m/s)	0.59 ± 0.19	0.96 ± 0.33	<0.001
GLS (%)	−15.91 ±0.20	−14.58 ±1.01	<0.001

*p*-value < 0.05 considered as significant.

## Data Availability

Data are available on request.
